# Integrative Genomic and Transcriptomic Analysis of White-Rot Fungi *Ganoderma tsugae* Growing on Both Coniferous and Broad-Leaved Trees

**DOI:** 10.3390/jof12010035

**Published:** 2026-01-01

**Authors:** Yifei Sun, Mengxue Lv, Meiqin Luo, Ziqi Yao, Miao Zhou, Yuxuan Fang, Dongmei Wu, Neng Gao, Baokai Cui

**Affiliations:** 1 State Key Laboratory of Efficient Production of Forest Resources, School of Ecology and Nature Conservation, Beijing Forestry University, Beijing 100083, China; 16603991133@163.com (M.L.); luomeiqin87@163.com (M.L.); yzq041122@163.com (Z.Y.); 13975796327@163.com (M.Z.); yuxuanfang2000@163.com (Y.F.); 2 Xinjiang Production and Construction Group Key Laboratory of Crop Germplasm Enhancement and Gene Resources Utilization, Biotechnology Research Institute, Xinjiang Academy of Agricultural and Reclamation Sciences, Shihezi 832000, China; wdm0993@163.com (D.W.); gaoneng520@163.com (N.G.)

**Keywords:** *Ganoderma*, White-rot fungi, whole-genome sequencing, transcriptome sequencing, differently expressed genes

## Abstract

*Ganoderma tsugae* is a typical white-rot fungus capable of decaying both coniferous and broad-leaved trees and is also used in traditional Chinese medicine for its immunomodulatory and anticancer properties. To elucidate the molecular basis of its broad substrate adaptability, we performed integrated genomic and transcriptomic analyses of two *G. tsugae* strains (collected from Xingjiang on *Betula* and Jilin on *Larix*). The high-quality genomes of *G. tsugae* Wu 2022 from Xinjiang (40.8 Mb, 12,496 genes) and *G. tsugae* Cui 14110 from Jilin (45.6 Mb, 13,450 genes) were obtained. There are enriched gene families related to carbohydrate-active enzymes (CAZymes) in two *G. tsugae* strains. Notably, specific CAZyme families implicated in hemicellulose (GH16), chitin metabolism (GH18), and ester bond cleavage (CE10) were prominently expanded. Transcriptome analyses under the induction of *Betula* and *Larix* sawdust revealed a core adaptive response. A total of 5558 genes were differentially expressed, including 2094 up-regulated and 3464 down-regulated genes. Most differentially expressed genes (DEGs) were annotated as “catalytic activity”, “metabolic processes” and specific functions such as nutrient transport (“MFS transporter”), and lipid metabolism (“3-oxoacyl-[acyl-carrier protein] reductase”). In addition, a conserved suite of the eleven shared DEGs were annotated as “Heat shock protein 9/12”, “alcohol dehydrogenase”, and “Cytochrome p450” related to secondary metabolites biosynthesis, transport, and catabolism. Based on the annotation results, the wood degradation mechanism of *G. tsugae* can be described as synthesizing and secreting degradation enzyme system to obtain energy, using protective enzyme systems to ensure its own health, and employing a transport enzyme system to recycle metabolic capacity. This progress ensures the environmental adaptability and high degradation efficiency of *G. tsugae* during wood degradation.

## 1. Introduction

*Ganoderma* P. Karst. (Polyporales, Ganodermataceae) comprises a group of white-rot fungi possessing lignocellulose-degrading capabilities [1], primarily mediated by an array of secreted enzymes including laccases, peroxidases, and dehydrogenases. The enzymatic machineries and underlying genetic basis of lignin degradation have been particularly well-characterized in models like *Phanerochaete chrysosporium* Burds., as well as in several *Ganoderma* species, revealing complex systems which involve numerous carbohydrate-active enzymes (CAZymes) [2,3,4], sugar metabolism pathways [5], and the overall degradation mechanisms [6]. The exploitation of these fungal systems extends beyond fundamental ecology into applied biotechnologies, including the bioremediation of aromatic pollutants [7,8,9], and the pretreatment of lignocellulosic biomass [8,10], underscoring their catalytic versatility. *Ganoderma tsugae* Murrill is not only a precious medicinal fungus in traditional Chinese medicine, with polysaccharides and triterpenoids valued for their immunomodulatory, antitumor, and anti-aging properties [11], but also a noteworthy model for studying substrate adaptability that could grow on both coniferous trees and broad-leaved trees [12]. It can cause white rot on *Tsuga*, *Larix,* and *Betula* in Northeast China and North America, posing a significant pathological threat [13] that suggests a robust and flexible degradative apparatus. Despite its dual importance in medicine and ecology, the specific genetic regulatory networks enabling *G. tsugae*’s broad host range wood decay remain largely unexplored. A preliminary assembly of *G. tsugae* (strain CCMJ4178, 43.26 Mb) provided a first appearance, identifying several genes (e.g., *salh*, *phea*, *cyp53a1*, *cyp102a*, *glpk*, and *amie*) potentially involved in plant–pathogen interaction, benzoate degradation, and fanconi anemia pathway [14].

Multi-omics approaches have been extensively applied to elucidate the lignocellulose-degradation mechanisms and secondary metabolism in white-rot fungi, including other *Ganoderma* species. The whole-genome sequencing and transcriptome analysis of *G. lucidum* (Curtis) P. Karst. have been conducted not only to investigate polysaccharide production under the influence of the reagent Tween 80, but also to identify a repertoire of genes encoding key lignin-modifying enzymes such as laccases and peroxidases [15]. Similarly, a systematic multi-omics study of *G. lingzhi* Sheng H. Wu, Y. Cao & Y.C. Dai identified over 500 carbohydrate active enzymes (CAZymes) and genes involved in recognition and biosynthesis of ganoderic acids, and a new special ganoderic acid A was also discovered [16]. Beyond the *Ganoderma* genus, integrated multi-omics frameworks such as genomics, transcriptomics, and proteomics have been successfully employed in wood decay fungi like *Phanerochaete chrysosporium* Burds., *Postia placenta* (Fr.) M.J. Larsen & Lombard, and *Wolfiporia cocos* (F.A. Wolf) Ryvarden & Gilb. to dissect the temporal regulation and synergistic action of complex enzyme systems during wood decay according to the different degradation strategies [17,18,19]. Additionally, CRISPR/Cas9-based gene editing and multi-omics integration (e.g., genomics, transcriptomics, and metabolomics) have been implemented in *G. lucidum* to optimize the production of bioactive compounds and to characterize gene networks underlying developmental processes [20,21]. Nevertheless, the genetic determinants of host-specific adaptation and wood degradation in *G. tsugae* have not been systematically explored using such approaches.

In this study, Illumina NovaSeq and PacBio sequencing technologies were used to sequence the high-quality genomes of two *Ganoderma tsugae* strains collected from Xinjiang Uygur Autonomous Region growing on *Betula* and Jilin Province growing on *Larix*. Based on the different culture media with *Betula* and *Larix* sawdust, the differentially expressed genes (DEGs) in the two *G. tsugae* strains were identified with transcriptomic analyses and completed the functional annotation using key function database. We will supplement comprehensive genomic and transcriptomic data for *G. tsugae*, explore the genetic characteristics of wood decomposition, and provide a molecular foundation for enhancing the efficiency of lignocellulose biodegradation and utilization. The purpose of this study is to elucidate the genetic and molecular mechanisms underlying the efficient lignocellulose degradation by *G tsugae*. Our findings will facilitate future research into the mechanistic basis of its degradation and utilization efficiency.

## 2. Materials and Methods

### 2.1. Fungal Strains and Culture Conditions

The experimental strains were isolated directly from fruiting bodies directly, in which the fruiting body of *Ganoderma tsugae* Wu 2022 was collected from Altay (47°50′ N, 88°39′ E), Xinjiang Uygur Autonomous Region, Northwest China, growing on *Betula*, while the fruiting body of *G. tsugae* Cui 14110 was collected from Lushuihe National Forest Park in Baishan (42°32′ N, 127°59′ E), Jilin Province, Northeast China, growing on *Larix*. They are deposited at the Institute of Microbiology, Beijing Forestry University, and are available upon request. The strains were preliminarily cultured on potato dextrose agar (PDA) medium in the dark at 25 °C for 7 days. Then, the genome sequencing was conducted on the mycelia of strains Wu 2022 and Cui 14110, which were cultured in liquid medium (malt extract medium, 2% malt powder, 1% glucose, and 0.3% KH_2_PO_4_) for 10 days at 25 °C in darkness. The mycelia were then collected and frozen in liquid nitrogen and stored at −80 °C.

Transcriptome sequencing was conducted on the mycelia growing on three carbon sources designated as *Betula* sawdust (B), *Larix* sawdust (L), and glucose (C). Glucose was used as a controlled experiment (following the liquid culture method as mentioned above). For the sawdust substrates media, 5 g of two kinds of wood sawdust in 40–60 mesh were placed in 125 mL Erlenmeyer flasks, respectively, to which 12.5 mL of deionized water was added. Then, 5 mL of mycelia homogenate was inoculated in each flask and cultured for 7 days at 25 °C. Each variable was repeated in triplicate to serve as parallel controls. The wood sawdust and Erlenmeyer flasks were sterilized by high-pressure steam and the inoculation and cultivation were carried out in a sterile environment.

### 2.2. Genome Sequencing, Assembly, and Annotation

The genomes of the two strains were *de novo* sequenced using high-throughput Illumina NovaSeq and PacBio sequencing platforms at Shanghai Personalbio Technology Co., Ltd. (Shanghai, China). The standard Illumina TruSeq Nano DNA LT library preparation protocol (Illumina TruSeq DNA Sample Preparation Guide) and the TruSeq DNA Sample Preparation Kit (Illumina, San Diego, CA, USA) were employed to construct genomic libraries for next-generation sequencing. The standard PacBio Template Preparation Kit 1.0 library preparation protocol (utilizing BluePippin for the selection of 20 kb templates) and the SMRTbell Template Preparation Kit 1.0 (Pacific Biosciences, California, USA) were utilized to create genomic libraries for third-generation sequencing. Prior to sequencing, library quality was assessed using the Agilent High Sensitivity DNA Kit on an Agilent Bioanalyzer. Following quality assessment, quantification of the libraries was carried out using the Quant-iT PicoGreen dsDNA Assay Kit (Thermo Fisher, Massachusetts, USA) on the Promega QuantiFluor fluorometric quantification system. To ensure the quality of subsequent data analyses, further filtration of the downstream data was conducted. Flye (https://github.com/fenderglass/Flye, accessed on 25 December 2025) and NextDenovo (https://github.com/Nextomics/NextDenovo, accessed on 25 December 2025) were used to perform *de novo* assembly of filtered reads to construct contigs and scaffolds, and the resulting assemblies were corrected in Pilon v.1.18 [22] to obtain the final genome sequence. Finally, BUSCO v.5.4.5 was used to assess the integrity of genome assembly [23].

The *de novo* prediction of genetic models, near-exotic species homology prediction, and transcriptome data-aided annotation methods were jointly used for the prediction of protein-coding genes [16]. Augustus v.2.5.5, glimmerHMM v.3.0.4, and GeneMark-ES v.4.71 were used for *de novo* prediction [24,25,26]. Exonerate v.2.20 was used for near-exotic species homology prediction. PASA v.2.4.1 was used to align transcripts to genomes for the structural annotation of genes. The sequence alignment of protein-coding genes was performed using DIAMOND v.2.0.14 [27]. The database utilized for the sequence alignment was the NCBI NR database v.4 (Non-Redundant Protein Sequence Database, http://ftp.ncbi.nih.gov/blast/db/, accessed on 25 December 2025). A significance threshold of 1e-6 was chosen for the sequence alignment, and the best hit was selected for functional annotation. The functional annotation of protein-coding genes was performed by utilizing BLAST v.2.5.0 [28] to compare against databases such as Non-redundant (NR) v.2017.10.10, Gene Ontology (GO) v.2023-01-01, Kyoto Encyclopedia of Genes and Genomes (KEGG), and Evolutionary Genealogy of Genes: Non-supervised Orthologous Groups (EggNOG). The GO annotation of protein-coding genes was achieved using Blast2go software (https://www.biobam.com/blast2go/, accessed on 25 December 2025) [29] referencing GO database v.105.1. The EggNOG annotation was performed using EggNOG-mapper v.2 [30], with the diamond alignment tool referencing the EggNOG database v.6.0 and a significance threshold set at 1 × 10^−6^. The primary objectives of KEGG annotation comprise the following two aspects: KO (KEGG Ortholog) annotation and KEGG v.105.1 Pathway annotation, which are primarily accomplished using KEGG Automatic Annotation System (KAAS) (https://www.genome.jp/kaas-bin/kaas_main, accessed on 25 December 2025) [31]. Furthermore, protein-coding genes were also annotated by blasting against Carbohydrate-Active Enzymes database (CAZy) v.2023, and hmmscan v.4.1 was used to predict the genes related to CAZymes in the genome sequence.

### 2.3. Transcriptome Sequencing and Annotation

Transcriptome sequencing for 18 mycelia samples was conducted on the Illumina NovaSeq sequencing platform by Shangh ai Personalbio Technology Co., Ltd. (Shanghai, China) and performed using *G. tsugae* CCMJ4178 (available in BioProject PRJNA733861, https://www.ncbi.nlm.nih.gov/datasets/genome/GCA_024305745.1/, accessed on 25 December 2025)) as reference genome-based read mapping. Total RNA was extracted using the Trizol Reagent (Invitrogen Life Technologies, CA, USA) following manufacturer’s protocol, then the concentration, quality, and integrity were determined using a NanoDrop spectrophotometer (Thermo Fisher, MA, USA). Clean reads were obtained after quality control using FASTQ v.0.22.0 [32]. The clean reads were then compared with a reference genome using HISAT2 v.2.1.0 (https://daehwankimlab.github.io/hisat2/, accessed on 25 December 2025) and used Trinity v.2.11.0 (https://github.com/trinityrnaseq/trinityrnaseq/issues/1131, accessed on 25 December 2025) for transcriptome assembly. Fragments Per Kilo bases per Million fragments (FPKM) was used to normalize the qualification of the expression levels. Based on FPKM expression data, the differential expression analysis was performed using DESeq2 v.1.38.3 R package [33]. Genes with an adjusted *p*-value < 0.05 and |log_2_(fold change)| > 2 were defined as differentially expressed genes (DEGs). DEGs were functionally annotated based on the GO v.2023-01-01, KEGG v.105.1, clusters of orthologous groups for eukaryotic complete genomes (KOG) database, and pathogen–host interactions (PHI) database.

## 3. Results

### 3.1. Whole-Genome Sequencing and Assembly

The genome information of *Ganoderma tsugae* Wu 2022 and *G. tsugae* Cui 14110 is shown in Table 1. The 1134 and 660 contigs were assembled into 1099 and 270 scaffolds forming 40.8 Mb genome and 45.6 Mb genome, with GC contents of 56.03% and 55.85% for *G. tsugae* Wu 2022 and *G. tsugae* Cui 14110, respectively. A total of 12,496 protein-coding genes were predicted in *G. tsugae* Wu 2022, with an average length about 1772 bp, and a total of 13,450 protein-coding genes were predicted in *G. tsugae* Cui 14110, with an average length of 1702 bp. The average exons per gene were 5.6 and 5.2 for the two strains, respectively. In addition, Benchmarking Universal Single-Copy Ortholog (BUSCO) assessment showed high completeness, with scores of 98.5% for *G. tsugae* Wu 2022 and 98.1% for *G. tsugae* Cui 14110, confirming high quality of the genome sequences for two strains in this study (Table 1).

### 3.2. Genome Functional Annotation

Protein-coding genes predicted in *Ganoderma tsugae* Wu 2022 and *G. tsugae* Cui 14110 were conducted to sequence similarity analysis against five databases to predict their potential functions. In general, the annotation profiles of the two strains were highly consistent, with no significant differences (Table 2). The annotation of NR database showed that 12,220 genes (97.8%) were annotated in *G. tsugae* Wu 2022 and 13,281 genes (98.7%) were annotated in *G. tsugae* Cui 14110. More than 90% of the genes in the two strains were matched with *Ganoderma* species (Supplementary Table S1). Through EggNOG database, most genes obtained annotation of “S: Function unknown” (3749 genes in *G. tsugae* Wu 2022 and 4007 genes in *G. tsugae* Cui 14110), and the subsequent frequent categories were “O: Posttranslational modification, protein turnover, chaperones” (609 genes in *G. tsugae* Wu 2022 and 620 genes in *G. tsugae* Cui 14110) and “G: Carbohydrate transport and metabolism” (561 genes in *G. tsugae* Wu 2022 and 596 genes in *G. tsugae* Cui 14110) (Supplementary Table S2).

**Figure 1:**
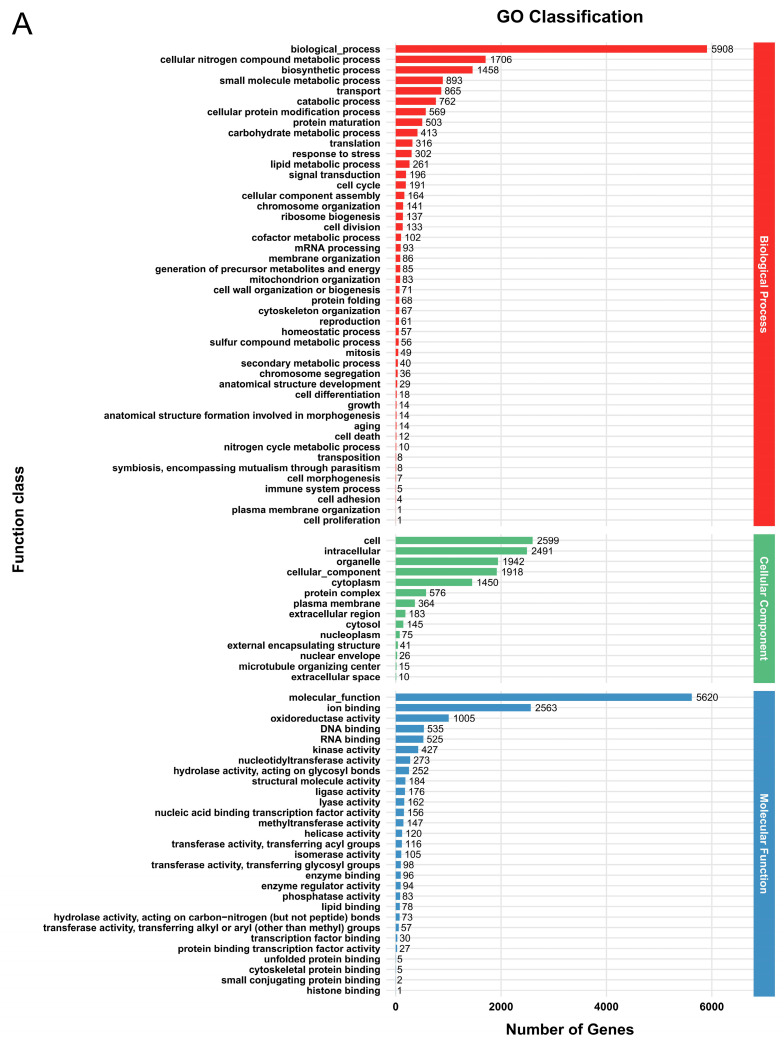

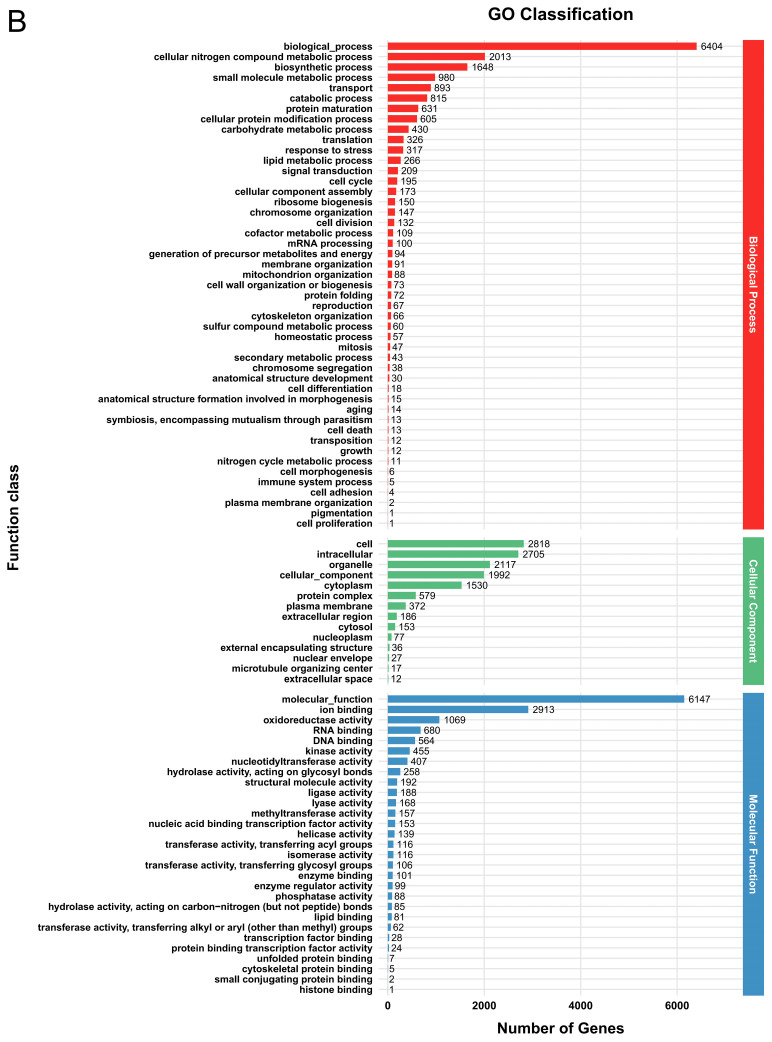
Functional annotation of predicted genes in two *Ganoderma tsugae* strains based on GO database. (**A**) *G. tsugae* Wu 2022. (**B**) *G. tsugae* Cui 14110. The different colors represent three function classes involving the related categories.

In the GO database, a total of 6616 genes (52.9%) in *G. tsugae* Wu 2022 and 7176 genes (53.4%) in *G. tsugae* Cui 14110 were matched into three functional classes with similar categories (Figure 1, Supplementary Table S3). In *G. tsugae* Wu 2022, most genes were annotated in the “biological process” category, i.e., biological processes (5908), cellular nitrogen compound metabolic process (1706), and biosynthetic processes (1458); cell (2599), intracellular (2491), and organelle (1942) in the “cell component” category; molecular function (5620), ion binding (2563), and oxidoreductase activity (1005) in the “molecular function” category (Figure 1A). Compared to *G. tsugae* Wu 2022, the GO enrichment analysis of *G. tsugae* Cui 14110 showed high consistency across categories with only differences in quantity (Figure 1B).

**Figure 2:**
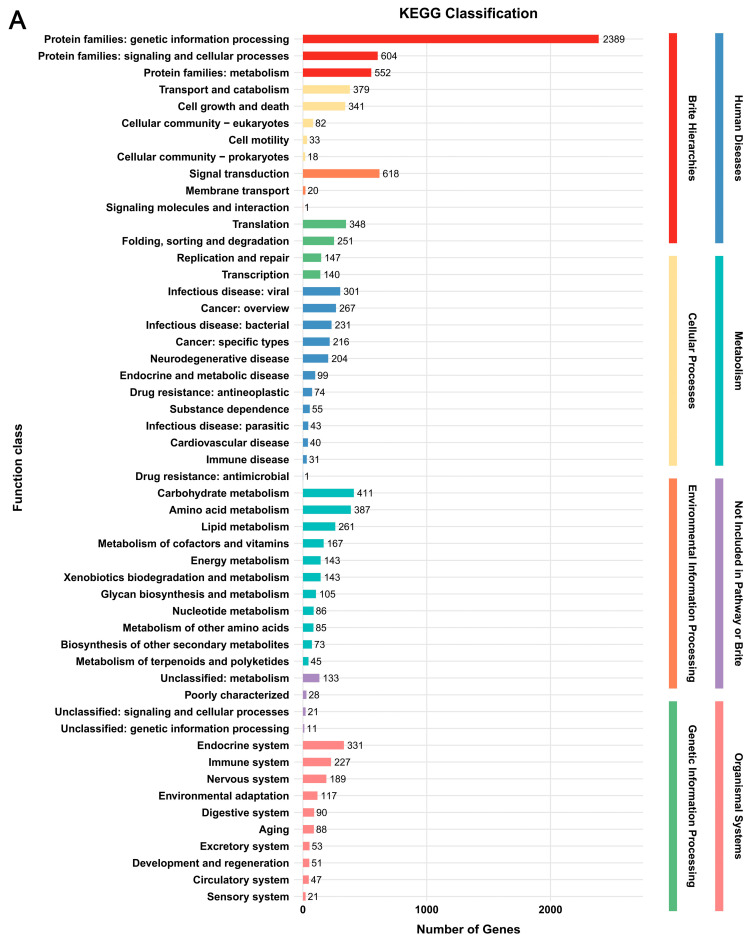

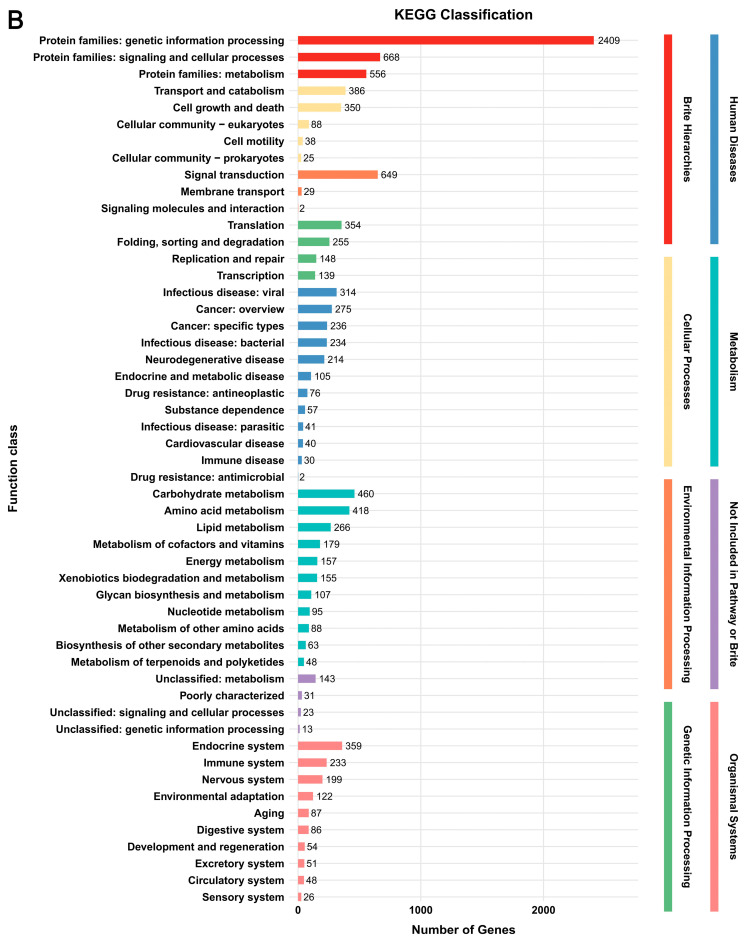
Functional annotation of predicted genes in two *Ganoderma tsugae* strains based on KEGG database. (**A**) *G. tsugae* Wu 2022. (**B**) *G. tsugae* Cui 14110. The different colors represent eight function classes involving the related categories.

In the KEGG database, a total of 3685 genes (29.4%) and 3824 genes (28.4%) were annotated, respectively (Figure 2, Supplementary Table S4). The predicted genes in the two strains were enriched in eight classes, i.e., Brite Hierarchies, Cellular Processes, Environmental Information Processing, Genetic Information Processing, Human Diseases, Metabolism, Not Included in Pathway or Brite, and Organismal Systems. The categories annotated in the two strains were highly consistent, with only quantitative differences. Among these categories, “Protein families: genetic information processing” in Brite Hierarchies had the highest number of genes (2389 genes in *G. tsugae* Wu 2022 and 2409 genes in *G. tsugae* Cui 14110). All other categories contained no more than 700 genes each, which together accounted for approximately 35%–37% of the total KEGG-annotated genes in each strain (Figure 2).

**Figure 3:**
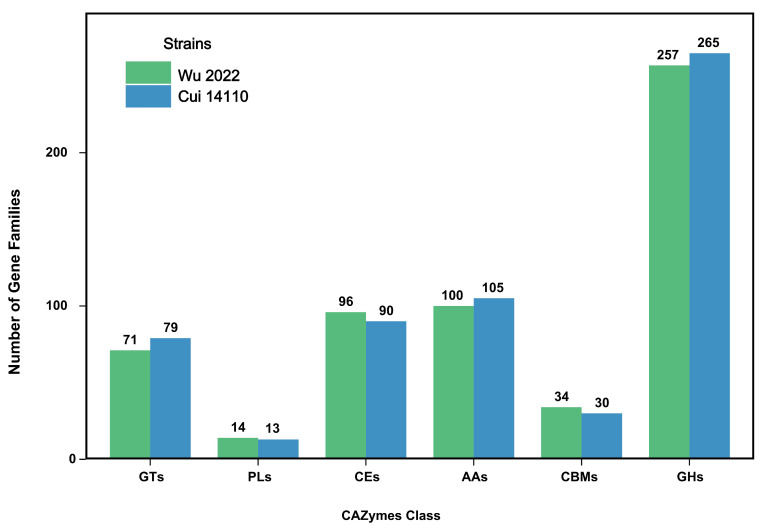
Functional annotation of predicted genes in two *Ganoderma tsugae* strains based on CAZymes database. GTs: glycosyl transferase; PLs: polysaccharide lyase; CEs: carbohydrate esterase; AAs: auxiliary activity; CBMs: carbohydrate-binding module; and GHs: glycoside hydrolase. The annotation results of *G. tsugae* Wu 2022 were in green, and those of *G. tsugae* Cui 14110 were in blue. The number of gene families for each class is shown above the plot.

A total of 572 gene families in *G. tsugae* Wu 2022 and 582 gene families in *G. tsugae* Cui 14110 were defined in the CAZymes (Figure 3, Supplementary Table S5). Notably, there was no significant difference (*p* > 0.05) in the quantity among six CAZymes classes. The gene families related to glycoside hydrolases (GHs) were most abundant in both strains (257 gene families in *G. tsugae* Wu 2022 and 265 gene families in *G. tsugae* Cui 14110), followed by auxiliary activities (AAs), carbohydrate esterases (CEs), glycosyl transferases (GTs), carbohydrate-binding modules (CBMs), and polysaccharide lyases (PLs). Both strains showed peak annotation in the CE10 (42, 39), GH16 (32, 35), and GH18 (35, 34).

### 3.3. Transcriptome Sequencing of G. Tsugae Cultured on Different Substrates

RNA sequencing was performed on the mycelia growing on glucose (C), *Betula* sawdust (B), and *Larix* sawdust (L). The mycelia of eighteen experimental samples for the two strains were collected for RNA-seq. After quality control, the clean reads against the reference genome ranged from 36.11 to 54.82 million reads for *G. tsugae* Wu 2022, and 42.14 to 50.07 million reads for *G. tsugae* Cui 14110. The mapping ratio of *G. tsugae* Wu 2022 ranged from 98.16% to 98.76%, and from 98.43% to 98.76% for *G. tsugae* Cui 14110. The Q20 value of each sample was above 98% (Supplementary Table S6).

**Figure 4:**
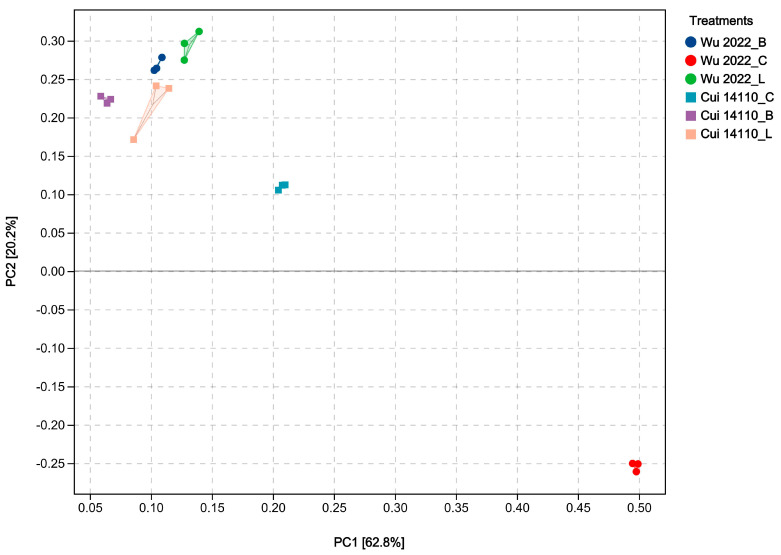
Principal component analysis (PCA) based on the FPKM standardized data of six treatments. The treatments labeled with circle and square are divided into *Ganoderma tsugae* Wu 2022 and *G. tsugae* Cui 14110 strains. The different colors represent the two strains growing on glucose medium (C), *Betula* sawdust (B), and *Larix* sawdust (L).

Principal component analysis (PCA) was conducted based on the FPKM standardized data from six treatments. In the two-dimensional PCA plot, the first two principal components (PC1 and PC2) explained 62.8% and 20.2% of the overall variance, respectively (Figure 4). It showed that six treatments formed distinct, separate clusters from each other. The glucose-grown treatments (Wu 2022_C and Cui 14110_C) showed a higher degree of intergroup dispersion compared to the other four treatments. Additionally, three experimental samples in the treatment of *G. tsugae* Cui 14110 growing on *Larix* sawdust (Cui 14110_L) exhibited higher degree of intragroup dispersion. Overall, the PCA demonstrated that the transcriptome sequencing data of six treatments was reliable for subsequent analyses.

### 3.4. Differential Expressed Genes Among Six Treatments

**Figure 5:**
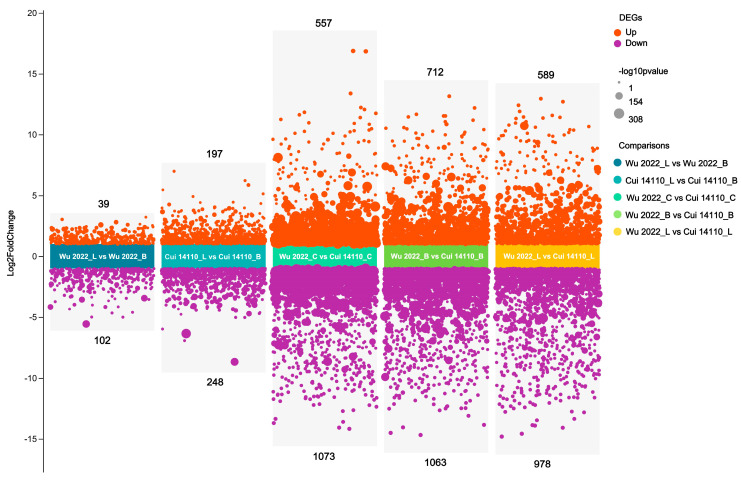
Number of DEGs among five comparisons. The number of up- and down-regulated genes for each category are shown above and below the plot.

Differential expressed genes (DEGs) were identified across the five comparisons using thresholds of |log_2_ Fold Change| > 2 and *p*-value < 0.05. A total of 5558 DEGs were found among five comparisons, including 2094 up-regulated and 3464 down-regulated genes. Among three inter-strain comparisons (Wu 2022_C vs. Cui 14110_C, Wu 2022_B vs. Cui 14110_B, and Wu 2022_L vs. Cui 14110_L), 4972 DEGs were identified, comprising 1858 up-regulated and 3114 down-regulated genes (Figure 5). In addition, there were 586 DEGs defined in two intra-strain, cross-substrate comparisons as follows: 445 DEGs in Wu 2022_L vs. Wu 2022_B and 141 DEGs in Cui 14110_L vs. Cui 14110_B, representing an approximately three-fold difference in the number of DEGs induced by substrate change between the two strains (Figure 5).

**Figure 6:**
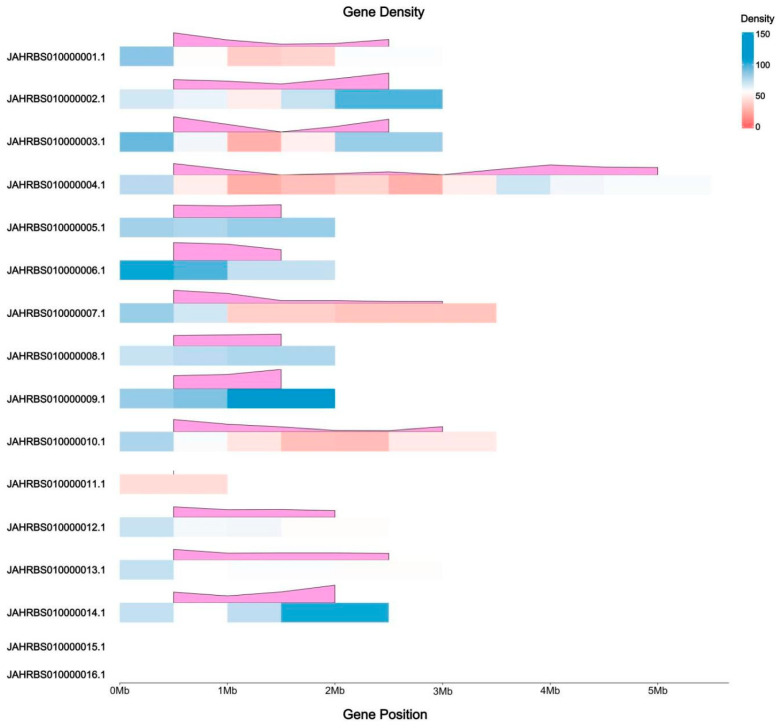
DEG density among five comparisons on reference genome (*Ganoderma tsugae* CCMJ4178). The colors from red to blue represent the number of DEGs, and the pink modules display the trend of DEG density.

The DEGs of the five comparisons were located on sixteen scaffolds of the reference genome (*Ganoderma tsugae* CCMJ4178), in which the length of DEGs on JAHRBS010000015.1 and JAHRBS010000016.1 were too short to show the density (Figure 6). The DEGs density of JAHRBS010000004.1 was highest accounting 9.9%, followed by JAHRBS010000009.1 (8.4%) and JAHRBS010000002.1 (8.2%). Almost no differentially expressed genes were located at JAHRBS010000011.1 (1.5%), JAHRBS010000015.1 (1.3%), and JAHRBS010000016.1 (0.4%). In addition, the trend of DEGs density in each scaffold is of no consistent pattern.

**Figure 7:**
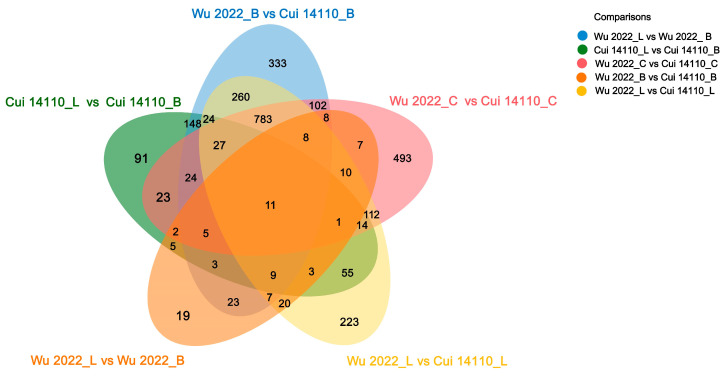
Venn diagram for shared and unique DEGs among five comparisons. The number of each module is shown on the plot.

The Venn diagram analysis showed 11 shared DEGs of five comparisons, and contained 333, 91, 19, 223, and 493 unique DEGs, respectively (Figure 7). Focusing on substrate effects, 300 DEGs were common to both strains when cultured on natural sawdust (L or B) compared to glucose (C). Within this set, thirty-nine genes were differentially expressed in both strains specifically when comparing the two sawdust types (L vs. B). There are 829 DEGs shared in the two strains under one kind of carbon sources designated as *Betula* sawdust (B), *Larix* sawdust (L), and glucose (C), respectively.

### 3.5. Functional Annotation of DEGs

**Figure 8:**
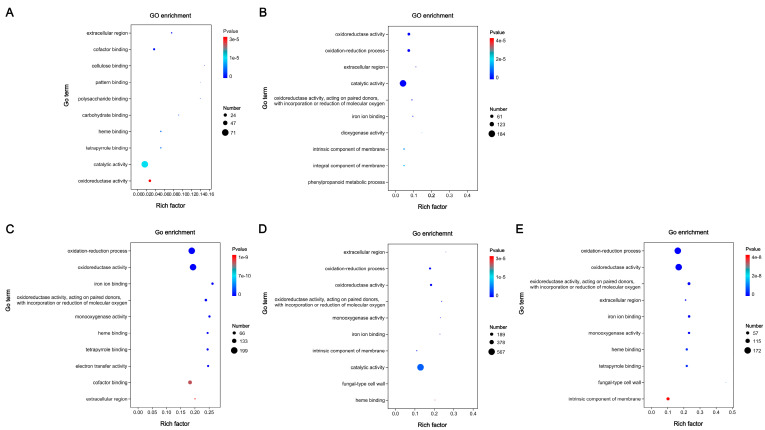
GO enrichment analyses of differentially expressed genes (DEGs) among five comparisons. (**A**) Wu 2022_L vs. Wu 2022_B. (**B**) Cui 14110_L vs. Cui 14110_B. (**C**) Wu 2022_C vs. Cui 14110_C. (**D**) Wu 2022_B vs. Cui 14110_B. (**E**) Wu 2022_L vs. Cui 14110_L.

**Figure 9:**
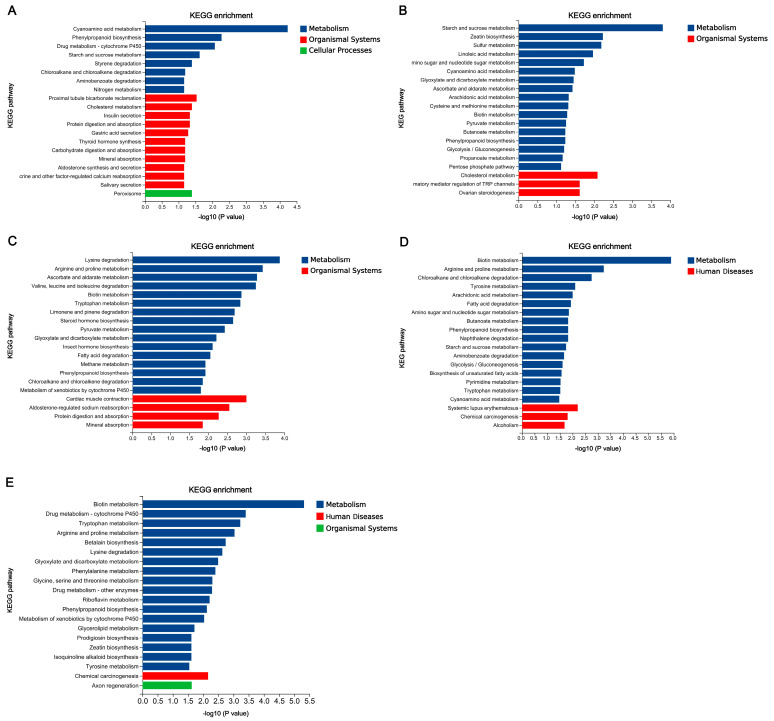
KEGG enrichment analyses of differentially expressed genes (DEGs) among five comparisons. (**A**) Wu 2022_L vs. Wu 2022_B. (**B**) Cui 14110_L vs. Cui 14110_B. (**C**) Wu 2022_C vs. Cui 14110_C. (**D**) Wu 2022_B vs. Cui 14110_B. (**E**) Wu 2022_L vs. Cui 14110_L.

The functional annotation of DEGs in five comparisons was conducted based on GO and KEGG database, respectively. There are 5936 GO terms annotated in five comparisons, including 460 non-redundant terms (Supplementary Table S7), and a total of 606 KEGG pathways were annotated among five comparisons, in which 54 terms were unique (Supplementary Table S8). The top ten GO terms and twenty KEGG pathways were shown in Figure 8 and Figure 9.

Based on GO enrichment analysis of the five comparisons (Figure 8), “catalytic activity”, “extracellular region”, and “oxidoreductase activity” were commonly enriched terms. All GO terms shown here were extremely significantly expressed (*p*-value < 0.00001). Most DEGs were annotated as category “catalytic activity” (GO: 0003824) in Wu 2022_L vs. Wu 2022_B (71), Cui 14110_L vs. Cui 14110_B (184), and Wu 2022_B vs. Cui 14110_B (567), while “oxidation-reduction process” (GO: 0055114) was the most annotated category in Wu 2022_C vs. Cui 14110_C (199) and Wu 2022_L vs. Cui 14110_L (172). In addition, the DEGs annotated as category “cellulose binding” (7), “dioxygenase activity” (13), “iron ion binding” (73), “extracellular region” (48), and “fungal-type cell wall” (1) were the least frequently annotated across the various comparisons.

According to the KEGG annotation results of five comparisons based on KEGG database (Figure 9), “Metabolism” was the common class annotated among five comparisons, and “Organismal Systems”, “Human Diseases”, and “Cellular Processes” also appeared in various comparisons. “Phenylpropanoid biosynthesis” was the only shared pathway in five comparisons. The annotated pathways in Wu 2022_L vs. Wu 2022_B clustered into three classes, and “Peroxisome” of “Cellular Processes” belonging to “Transport and catabolism” was unique. The most pathways annotated in each comparison were various, such as “Cyanoamino acid metabolism” belonging to “Metabolism of other amino acids”, “Starch and sucrose metabolism” belonging to “Carbohydrate metabolism”, “Lysine degradation” belonging to “Amino acid metabolism”, and “Biotin metabolism” belonging to “Metabolism of cofactors and vitamins”. The pathways grouped in “Human Diseases” were annotated in Wu 2022_B vs. Cui 14110_B and Wu 2022_L vs. Cui 14110_L, which were related to “Systemic lupus erythematosus”, “Chemical carcinogenesis”, and “Alcoholism”.

The comparisons of Wu 2022_C vs. Cui 14110_C, Wu 2022_B vs. Cui 14110_B, and Wu 2022_L vs. Cui 14110_L shared 829 DEGs, which were mostly annotated as GO: 0008152 (252, metabolic process) belonging to class “Biological Process (BP)” in GO database and K00652 (8-amino-7-oxononanoate synthase) in KEGG database. Within this shared set, 300 DEGs were common specifically to Wu 2022_B vs. Cui 14110_B and Wu 2022_L vs. Cui 14110_L, but not to Wu 2022_C vs. Cui 14110_C. These DEGs were also mostly annotated as GO: 0008152 (252, metabolic process) belonging to class “Biological Process (BP)” in GO database, and K08192 (MFS transporter, ACS family, DAL5 transporter family protein) and K00059 (3-oxoacyl-[acyl-carrier protein] reductase) in KEGG database (Supplementary Table S9).

The comparisons of Wu 2022_L vs. Wu 2022_B and Cui 14110_L vs. Cui 14110_B shared 39 DEGs induced by two kinds of sawdust (Supplementary Table S10). Besides GO: 0003824 (18, catalytic activity), most DEGs were enriched in the GO database as GO: 0008152 (18, metabolic process) belonging to class “Biological Process (BP)”. Three KEGG pathways were annotated, such as K17069 (O-acetylhomoserine/O-acetylserine sulfhydrylase), K01539 (sodium/potassium-transporting ATPase subunit alpha), and K13050 (proprotein convertase subtilisin/kexin type 9). PHI: 2393 (O-methylsterigmatocystin oxidoreductase) was annotated for a small number of DEGs in Wu 2022_L vs. Wu 2022_B and Cui 14110_L vs. Cui 14110_B. There were 19 unique DEGs in Wu 2022_L vs. Wu 2022_B (14 down-regulated and 5 up-regulated) and 91 DEGs in Cui 14110_L vs. Cui 14110_B (40 down-regulated and 51 up-regulated) (Supplementary Table S11). Besides the common GO: 0003824 (catalytic activity) and GO: 0008152 (metabolic process), the most common ones were GO: 0005488 (11, binding) belonging to class “Molecular Functions (MF)”, and GO: 0009987 (23, cellular process) belonging to class “Biological Process (BP)”, respectively. Two KEGG pathways were annotated as “nitrilase” and “cytochrome P450 family 13”.

The eleven DEGs shared by five comparisons were located at scaffolds JAHRBS010000001.1, JAHRBS010000002.1, JAHRBS010000003.1, JAHRBS010000004.1, JAHRBS010000006.1, JAHRBS010000009.1, JAHRBS0100000013.1, and JAHRBS0100000014.1, with gene lengths ranging from 252 to 1713 bp (Supplementary Table S12). Most DEGs were annotated as GO: 0003824 (7, catalytic activity) belonging to class “Molecular Functions (MF)”, and GO: 0008152 (7, metabolic process) belonging to class “Biological Process (BP)”. In KOG database, class “Q: Secondary metabolites biosynthesis, transport and catabolism” was the most noticeable, which was annotated as category “Heat shock protein 9/12”, “alcohol dehydrogenase”, and “Cytochrome p450”. There were no DEGs annotated in KEGG database.

## 4. Discussion

Through whole-genome sequencing of the two strains of *Ganoderma tsugae*, we obtained a high-quality genome assembly for each strain. The strain Wu 2022 from Xinjiang has a genome size of 40.8 Mb encoding 12,496 protein-coding genes, while the strain Cui 14110 from Jilin has a genome of 45.6 Mb with 13,450 protein-coding genes (Table 1). These genome sizes and gene counts are comparable to, though slightly divergent from, a previously published *G. tsugae genome* (strain CCMJ4178, 18 contigs, and 10,946 protein-coding genes) [14]. The observed variations in genome size and gene content among the two strains likely reflect both natural genomic diversity between different *G. tsugae* isolates and technical discrepancies arising from the use of different sequencing platforms and assembly methodologies. Until now, there have been twenty-six published genomes of *Ganoderma* presenting sixteen species on JGI (https://mycocosm.jgi.doe.gov/fungi/fungi.info.html, accessed on 25 December 2025) and NCBI (https://www.ncbi.nlm.nih.gov/, accessed on 25 December 2025). Reported genomes sizes for *Ganoderma* species range from 39 to 92 Mb, and the three genomes of *G. tsugae* are smaller than most species, especially the largest genome of *G. adspersum* CBS 147723 (https://genome.jgi.doe.gov/portal/GanadsStandDraft_FD/, accessed on 25 December 2025).

According to the functional annotation of protein-coding genes from *Ganoderma tsugae* Wu 2022 and *G. tsugae* Cui 14110 across five databases, the two strains exhibit highly conserved functional profiles, with no significant differences observed in overall annotation categories (Table 2). It indicated that despite their distinct geographical origins and plant hosts, both strains retain core molecular mechanisms characteristic of *G. tsugae*. In NR database, *G. leucocontextum* was the most frequently annotated species, which always formed sister branches with *G. tsugae* in phylogenetic analysis [1]. The shared gene families and functional modules are also worth considering, especially the part related to conserved lignocellulolytic mechanisms. The functional profiles of *G. tsugae* Wu 2022 and *G. tsugae* Cui 14110 in the EggNOG database indicate that the related proteins are modified or pathways are activated to initiate protein synthesis and recovery through protein phosphorylation [34,35] and ubiquitination [36,37], thereby secreting carbohydrate hydrolases to degrade lignocellulose [38]. The most abundant term in GO database is “biological processes”, and the predominantly KEGG pathway is “Brite Hierarchies” concerning “Protein families: genetic information processing” (Figure 2). It implies that the proteins encoded by their genomes are involved in extremely diverse and active cellular activities [39]. Hierarchy directly points to the central law of life: DNA to RNA to protein [40]. Its enrichment means that the genome of the two *G. tsugae* strains has invested a significant number of resources in the expression of genetic information [41]. In addition, the two strains were also annotated multiple carbohydrate metabolism pathways, including “Starch and sucrose metabolism”, “Galactose metabolism”, “Fructose and mannose metabolism”, etc., and pathways related to protein synthesis, modification, transport, and secretion associated with hydrolase secretion (Table S4). In contrast, the difference between the two is that *G. tsugae* Wu 2022 may have higher protein synthesis efficiency, and *G. tsugae* Cui 14110 has more advantages in environmental adaptation and signal response, which may affect the secretion regulation and functional expression of its hydrolytic enzymes.

Functional annotation based on CAZymes database revealed a highly similar performance of carbohydrate-active enzymes between *Ganoderma tsugae* Wu 2022 and *G. tsugae* Cui 14110, with abundant glycoside hydrolases (GHs) (Figure 3). Cellulose and hemicellulose, the main components of wood, are both polysaccharides [42]. Glycoside hydrolases (GHs) specifically catalyze the hydrolysis of glycosidic bonds in these polysaccharide chains [43,44]. GH16 and GH18 families were enriched (Supplementary Table S5), and the xyloglucanase/TETase in the former one can cleave the main chain of xyloglucan and loosen the cellulose hemicellulose network [45], while the chitinase in the latter helps hyphae grow and branch to continuously reshape its cell wall during the penetration of hard wood [46,47]. The number of gene families annotated as carbohydrate esterases (CEs) were smaller than those annotated as GHs and auxiliary activities (AAs), but the CE10 family had the highest number among all carbohydrate annotation categories (Supplementary Table S5). CE10 is a multifunctional esterase family and it has a very wide substrate spectrum [48]. The function of this family includes degradation of side chains of hemicellulose, cutting off the bonds of lignin-carbohydrate complexes (LCCs) and breaking down plant defense compounds [49,50]. We speculate that wood degradation by *G. tsugae* follows a sequential enzymatic strategy as follows: abundant CE10 enzymes unbind and loosen LCCs and hemicellulose side chains; then, GH16 enzymes extensively attack hemicellulose, while GH18 chitinase supports sustained hyphal growth; finally, other GHs and AAs enzymes further degrade the exposed cellulose and lignin.

The two *Ganoderma tsugae* strains used in this study were collected from different trees, i.e., Wu 2022 growing on *Betula* in the Altay region, Xinjiang, and Cui 14110 growing on *Larix* in the Lushuihe region, Jilin. Principal component analysis (PCA) of their transcriptional profiles under three culture substrates showed clear separability, reflecting a strong substrate-driven influence on gene expression (Figure 4). The dispersion of the Wu2022_C and Cui 14110_C cannot be ignored; it may suggest that this artificial substrate introduces distinct physiological or stress responses compared to natural sawdust, a pattern further supported by differential gene expression (DEG) analysis (Figure 5). Wu2022_C and Cui 14110_C showed 557 up-regulated and 1073 down-regulated DEGs, including 493 unique DEGs (Figure 7). Compared to it, when the strains induced under two kinds of natural sawdust, Wu 2022_B vs. Cui 14110_B and Wu 2022_L vs. Cui 14110_L) presented 300 shared DEGs (Supplementary Table S9). Functional annotation of these shared DEGs highlighted enrichment in metabolic processes (GO:0008152), transmembrane transport (K08192, MFS transporter), and lipid metabolism (K00059, 3-oxoacyl-ACP reductase). It indicates that faced with more complex and nutritionally imbalanced culture media with natural sawdust, *G. tsugae* may induce the expression of more genes related to metabolism. The purpose may be to degrade cellulose and hemicellulose to obtain energy [51], or to synthesize secondary metabolites such as triterpenes to protect itself from harm [52]. There are unexpectedly few DEGs when comparing the same strain on *Larix* or *Betula* (Wu 2022_L vs. Wu 2022_B and Cui 14110_L vs. Cui 14110_B), especially in the *G. tsugae* Wu 2022. It revealed that the inter-strain differences are greater than those induced by sawdust, suggesting the strains have essential differences in genetic background, physiological status, or response to environment/treatment.

The comparisons of Wu 2022_L vs. Wu 2022_B and Cui 14110_L vs. Cui 14110_B shared thirty-nine DEGs induced by two kinds of sawdust (Supplementary Table S10). The enrichment of these shared genes highlights a conserved transcriptional response essential for substrate adaptation. Most DEGs were annotated as catalytic activity (GO:0003824), which can enhance enzyme activity related to wood degradation [53], such as laccase, endoglucanase, acetylesterase, and so on [54,55]. This conserved induction underscores a fundamental strategy in *G. tsugae* to optimize its lignocellulolytic system when encountering natural woody substrates. PHI: 2393 (O-methylsterigmatocystin oxidoreductase) was noticed in Wu 2022_L vs. Wu 2022_B and Cui 14110_L vs. Cui 14110_B; it is one of the key enzymes for synthesizing aflatoxins [56]. However, for medicinal fungi, O-methylsterigmatocystin oxidoreductase may be involved in the activity of lignin-degrading enzymes such as lignin peroxidase or manganese peroxidase rather than synthesize toxins [57].

Among five comparisons, the distinct but interpretable patterns emerged in the functional annotation of DEGs that reflect both the conserved lifestyle and adaptive strategies of *Ganoderma tsugae* (Figure 9). The “catalytic activity”, “extracellular region”, and “oxidoreductase activity” of GO enrichment analysis were consistently enriched in five comparisons (Figure 8). This recurring profile aligns with the core functional signature of white-rot fungi, fundamentally shaped by their saprotrophic lifestyle—requiring extensive secretion of extracellular enzymes, redox balancing, and active energy metabolism to degrade lignocellulose [58,59,60]. Under KEGG annotation, most DEGs were related to class “Metabolism”, and “Phenylpropanoid biosynthesis” was the only shared pathway in five comparisons (Figure 9). Unlike plants, which synthesize lignin monomers through phenylpropanoid biosynthesis [61], *G. tsugae* has a large enzyme system capable of decomposing and metabolizing phenylpropane derivatives. There are eleven DEGs shared by five comparisons, regardless of the changes in strains or culture media. These DEGs were located across multiple contigs (JAHRBS010000001.1, JAHRBS010000002.1, JAHRBS010000003.1, JAHRBS010000004.1, JAHRBS010000006.1, JAHRBS010000009.1, JAHRBS0100000013.1, and JAHRBS0100000014.1), which accommodated a series of terpene synthase-related gene clusters in the reference genome (*Ganoderma tsugae* CCMJ4178) [14]. This genomic colocalization suggests that these conserved responses may be embedded within broader secondary metabolite regions, potentially coordinating chemical defense with substrate degradation. It is worth noting that “Heat shock protein 9/12”, “alcohol dehydrogenase”, and “Cytochrome p450” belong to class “Q: Secondary metabolites biosynthesis, transport and catabolism”, endowing these genes with powerful functions based on the annotation of KOG database. Cytochrome p450 contributes to efficient lignocellulose degradation [62]; on the other hand, heat shock proteins help maintain protein structure and functional stability [63]; additionally, alcohol dehydrogenase aids in recovering degradation products [64]. Together, these conserved genes form a robust functional triad that enables *G. tsugae* to sustain efficient lignocellulose degradation while managing cellular stress and metabolic byproducts across varied environments.

Currently, artificial cultivation of *Ganoderma tsugae* has been achieved, and the differences in nutrient composition, cultural conditions, and strains sources can all affect the degradation and growth rates of *G. tsugae*. In this study, the genome and transcriptome analyses were conducted to preliminarily explore the genetic characteristics of *G. tsugae* induced by different culture media. It indicates that there are the following three types of significantly expressed genes in *G. tsugae* during the process of degrading wood: attack type degradation enzyme system, protective type defense enzyme system, and recycling type transport enzyme system. The synergistic operation of these enzymes enables *G. tsugae* to have strong environmental adaptability and ecological competitiveness, enabling it to survive and reproduce on both coniferous and broad-leaved trees. These findings help us gain a deeper understanding of the unique wood degradation mechanism of *G. tsugae*. 

## 5. Conclusions

This study preliminarily elucidates the synergistic molecular mechanism of lignocellulose degradation in *Ganoderma tsugae* based on genomic and transcriptomic analyses. The high-quality genomes and comparative transcriptomes of two strains adapted to different hosts for *G. tsugae* were supplemented. Our results reveal that the fungus mobilizes a coordinated network involving degradative enzyme production, cellular protection, and secondary metabolite turnover to efficiently adapt to and deconstruct complex lignocellulosic matrices. This integrated strategy underpins its broad host range and high degradation efficacy on both coniferous and broad-leaved wood. Future investigations should focus on the functional validation of key regulatory genes and incorporation of proteomic and metabolomics data to validate enzyme secretion, track lignin degradation products, and perform functional validation on key candidate genes.

## Figures and Tables

**Table 1:** Genome information of two strains of *Ganoderma tsugae*.

Parameter	*G. tsugae* Wu 2022	*G. tsugae* Cui 14110
Sequencing platform	Illumina NovaSeq	Illumina NovaSeq pacbio Sequel
Genome size (Mb)	40.8	45.6
Number of contigs	1134	660
Contig N50 (bp)	100,812	168,103
Number of scaffolds	1099	270
Scaffold N50 (bp)	103,400	3,119,812
Number of genes	12,496	13,450
Average gene length (bp)	1772	1702
Average exons per gene	5.6	5.2
GC content (%)	56.03	55.85
BUSCO (% complete)	98.5	98.1

**Table 2:** Functional annotation of predicted genes in two *Ganoderma tsugae* strains based on five databases.

Database	*G. tsugae* Wu 2022	*G. tsugae* Cui 14110
NR	12,220 (97.8%)	13,281 (98.7%)
eggNOG	9203 (73.6%)	9835 (73.1%)
GO	6616 (52.9%)	7176 (53.4%)
KEGG	3685 (29.4%)	3824 (28.4%)
CAZymes	572 (4.58%)	582 (4.38%)

Note: the percentage values in parentheses represent the proportion of annotated genes in the total number of protein-coding genes.

## Data Availability

All data generated or analyzed for this study are included in this article and its supplementary materials (Tables S1–S12). The raw sequence data reported in this paper have been deposited in the Genome Sequence Archive [65] in National Genomics Data Center [66], China National Center for Bioinformation/Beijing Institute of Genomics, and Chinese Academy of Sciences (GSA: CRA033453 and CRA034265), all of which are publicly accessible at https://ngdc.cncb.ac.cn/gsa (accessed on 25 December 2025).

## Supplementary Materials

The following supporting information can be downloaded at: https://www.mdpi.com/article/10.3390/jof12010035/s1, Table S1: Annotation of two strains on NR database, Table S2: Annotation of two strains on eggNOG database, Table S3: Annotation of two strains on GO database, Table S4: Annotation of two strains on KEGG database, Table S5: Annotation of two strains on CAZymes database, Table S6: Transcriptional sequencing data and quality assessment, Table S7. GO enrichment analyses of DEGs among five comparisons, Table S8: KEGG enrichment analyses of DEGs among five comparisons, Table S9: Functional annotation information of 829 DEGs of Wu 2022_C vs. Cui 14110_C, Wu 2022_B vs. Cui 14110_B and Wu 2022_L vs. Cui 14110_L, Table S10: Functional annotation information of 39 shared DEGs of Wu 2022_L vs. Wu 2022_B and Cui 14110_L vs. Cui 14110_B, Table S11: Functional annotation information of unique DEGs of Wu 2022_L vs. Wu 2022_B and Cui 14110_L vs. Cui 14110_B, Table S12: Functional annotation information of eleven DEGs of five comparisons.
